# Valproic acid silencing of *ascl1b/Ascl1* results in the failure of serotonergic differentiation in a zebrafish model of fetal valproate syndrome

**DOI:** 10.1242/dmm.013219

**Published:** 2013-10-17

**Authors:** John Jacob, Vanessa Ribes, Steven Moore, Sean C. Constable, Noriaki Sasai, Sebastian S. Gerety, Darren J. Martin, Chris P. Sergeant, David G. Wilkinson, James Briscoe

**Affiliations:** 1 Division of Developmental Biology, MRC National Institute for Medical Research, The Ridgeway, Mill Hill, London, NW7 1AA, UK.; 2 National Hospital for Neurology and Neurosurgery, Queen Square, London, WC1N 3BG, UK.; 3 Division of Developmental Neurobiology, MRC National Institute for Medical Research, The Ridgeway, Mill Hill, London, NW7 1AA, UK.; 4 London Research Institute, Cancer Research UK, Lincoln’s Inn Fields Laboratories, 44 Lincoln’s Inn Fields, London, WC2A 3LY, UK.

**Keywords:** Serotonin, Fetal valproate syndrome, Zebrafish, Notch, Proneural gene, Hdac1

## Abstract

Fetal valproate syndrome (FVS) is caused by *in utero* exposure to the drug sodium valproate. Valproate is used worldwide for the treatment of epilepsy, as a mood stabiliser and for its pain-relieving properties. In addition to birth defects, FVS is associated with an increased risk of autism spectrum disorder (ASD), which is characterised by abnormal behaviours. Valproate perturbs multiple biochemical pathways and alters gene expression through its inhibition of histone deacetylases. Which, if any, of these mechanisms is relevant to the genesis of its behavioural side effects is unclear. Neuroanatomical changes associated with FVS have been reported and, among these, altered serotonergic neuronal differentiation is a consistent finding. Altered serotonin homeostasis is also associated with autism. Here we have used a chemical-genetics approach to investigate the underlying molecular defect in a zebrafish FVS model. Valproate causes the selective failure of zebrafish central serotonin expression. It does so by downregulating the proneural gene *ascl1b*, an ortholog of mammalian *Ascl1*, which is a known determinant of serotonergic identity in the mammalian brainstem. *ascl1b* is sufficient to rescue serotonin expression in valproate-treated embryos. Chemical and genetic blockade of the histone deacetylase Hdac1 downregulates *ascl1b*, consistent with the Hdac1-mediated silencing of *ascl1b* expression by valproate. Moreover, tonic Notch signalling is crucial for *ascl1b* repression by valproate. Concomitant blockade of Notch signalling restores *ascl1b* expression and serotonin expression in both valproate-exposed and *hdac1* mutant embryos. Together, these data provide a molecular explanation for serotonergic defects in FVS and highlight an epigenetic mechanism for genome-environment interaction in disease.

## INTRODUCTION

Valproate (VPA) is a fatty acid derivative widely prescribed for its anticonvulsant, mood-stabilising and pain-relieving properties, but it has teratogenic and neuropsychiatric side effects upon *in utero* exposure, collectively termed fetal valproate syndrome (FVS). The underlying molecular cause of FVS is unknown, but candidate mechanisms are the dysregulation of transcription factors important for brain development, disruption of signal transduction pathways, inositol depletion and direct inhibition of epigenetic regulators such as the histone deacetylases (HDACs) (Chen et al., 1997; Detich et al., 2003; Einat et al., 2003; Marchion et al., 2005; Milutinovic et al., 2007; Phiel et al., 2001; Williams et al., 2002).

Fetal VPA exposure is associated with a 3- to 46-fold increased risk of autism spectrum disorder (ASD) (Bromley et al., 2013; Christensen et al., 2013; Dufour-Rainfray et al., 2011; Rasalam et al., 2005). Animal models of FVS display autism-like behaviours (Dufour-Rainfray et al., 2010; Kim et al., 2011; Yochum et al., 2008) and neuroanatomical abnormalities that are also reported in ASD (Ingram et al., 2000; Rodier et al., 1996). In these models, activity of the neurotransmitter serotonin (5HT) is altered, which has been implicated in the regulation of numerous behaviours, including social interaction (Ansorge et al., 2004; Patterson, 2006). Altered hippocampal and blood 5HT levels have been reported in animal models of FVS, which correlate with impaired 5HT neuronal differentiation (Dufour-Rainfray et al., 2010; Kuwagata et al., 2009; Miyazaki et al., 2005; Narita et al., 2002; Oyabu et al., 2013) and autism-like behaviours (Lin et al., 2013; Tsujino et al., 2007; Wang et al., 2013). Interestingly, in one of these rat models, treatment with a 5HT_1A_ receptor agonist improved the abnormal behaviours, implying a deficit of 5HT signalling (Wang et al., 2013). By contrast, in the other study, VPA increased brain 5HT levels (Tsujino et al., 2007).

Significantly, 5HT is also implicated in autism pathogenesis. In ASD, 30% of subjects have elevated 5HT blood levels (Mulder et al., 2004; Schain and Freedman, 1961), central 5HT homeostasis is altered (Chugani et al., 1999; Chugani et al., 1997) and an association with stereotyped behaviour has been reported (Kolevzon et al., 2010; Sacco et al., 2010). Selective serotonin reuptake inhibitors (SSRIs) improve some manifestations of autism, including stereotypical behaviours (Hollander et al., 2003; McDougle et al., 2000), whereas depletion of the 5HT precursor tryptophan exacerbates these symptoms (Bauman et al., 2006). Genetic or pharmacological perturbation of the 5HT system is associated with autism-like behaviours in humans and in rodents (Bauman et al., 2006; Cook et al., 1997; Kane et al., 2012; Klauck et al., 1997; Nabi et al., 2004; Nakatani et al., 2009; Sutcliffe et al., 2005; Veenstra-VanderWeele et al., 2012). In particular, an allelic polymorphism of the serotonin transporter gene (*SERT*), which is a determinant of 5HT activity, is associated with ASD (Devlin et al., 2005). Furthermore, a mouse model of one of the human gain-of-function *SERT* genetic variants displays ASD-like behaviours and hyperserotonaemia (Veenstra-VanderWeele et al., 2012). Therefore, increases and decreases in central 5HT activity seem to produce common behavioural phenotypes, which is consistent with the view that autism can result from positive and negative changes in neurotransmitter signalling (Zoghbi and Bear, 2012).

**TRANSLATIONAL IMPACT**

**Clinical issue**

The drug valproate is used worldwide as an anticonvulsant agent, as a mood stabiliser and for its pain-relieving properties. Valproate is teratogenic (interferes with early development) and fetal exposure causes fetal valproate syndrome (FVS), which is characterised by a spectrum of morphological, cognitive and behavioural deficits. Recent population-based epidemiological studies have highlighted the significantly increased risk of autism spectrum disorders (ASDs) in children exposed to valproate *in utero*. The *in vivo* mechanism of valproate action that is pertinent to its neuropsychiatric side effects is not clear. Multiple *in vitro* mechanisms have been described, including inhibition of histone deacetylases. Studies on animal models of FVS have identified biochemical and cellular perturbations of the central serotonergic system. Altered serotonin homeostasis is also a feature of idiopathic autism; therefore, uncovering the pathogenesis of serotonin deficits in FVS could reveal the molecular underpinnings of core behavioural abnormalities in autism.

**Results**

Zebrafish are highly suited to pharmacological and genetics approaches that can be combined to provide novel insights into disease mechanisms. In this article, the authors describe a zebrafish model of FVS that displays a failure of serotonergic differentiation in the brainstem in response to valproate treatment. They show that a critical proneural gene, *ascl1b*, is silenced by valproate through a mechanism that depends on inhibition of the histone deacetylase Hdac1. Their experiments further show that valproate unmasks tonic repression of the *ascl1b* gene by the Notch pathway. If the Notch pathway is blocked, valproate is no longer able to silence *ascl1b*. Importantly, restoration of Ascl1b in the presence of valproate rescues the expression of serotonin in the zebrafish brainstem.

**Implications and future directions**

This study shows that valproate represses expression of *ascl1b*, leading to defects in the serotonergic system in zebrafish. The failure in serotonergic differentiation in this new model is reminiscent of defects reported previously for mice exposed to valproate, suggesting that the mechanism unveiled herein is likely to provide a common molecular explanation for serotonergic abnormalities in this disorder. Indeed, the conservation of the differentiation pathways of serotonergic neurons in zebrafish and in humans suggests that these findings will be relevant to understanding the complex pathophysiology of FVS. More broadly, the authors highlight an epigenetic mechanism at work in an iatrogenic form of ASD that could also be relevant to idiopathic, common forms of autism.

5HT neurons in the hindbrain are derived from progenitors exposed to the signalling molecule sonic hedgehog (Shh) (Jessell, 2000). Serotonergic progenitor identity is characterised by expression of the transcription factors Nkx2.2, Foxa2 and Ascl1, all of which are required for 5HT neuronal differentiation (Briscoe et al., 1999; Jacob et al., 2007; Pattyn et al., 2004). Newly born 5HT neurons express post-mitotic determinants, including the transcription factor Pet1 (Hendricks et al., 2003) and subsequently the 5HT biosynthetic enzyme Tph2 (Zhang et al., 2004).

We investigated the molecular pathophysiology underlying serotonergic deficits in a zebrafish FVS model because this could provide mechanistic insight into the genesis of core autism behaviours. Importantly, hindbrain development and neuronal subtype diversity and serotonergic differentiation show strong conservation (Lillesaar, 2011). Furthermore, zebrafish exposed to VPA display morphological defects similar to those described in FVS, suggesting the validity of our system for modelling features of human FVS (Gurvich et al., 2005; Herrmann, 1993). We show that VPA specifically blocks hindbrain 5HT expression in zebrafish. Acting via Hdac1, VPA silences the zebrafish ortholog of mammalian *Ascl1*, *ascl1b*, by unmasking tonic Notch repression. Moreover, Ascl1b is sufficient to rescue 5HT expression in VPA-treated embryos.

## RESULTS

### VPA impairs central 5HT neuronal differentiation

**Fig. 1.**
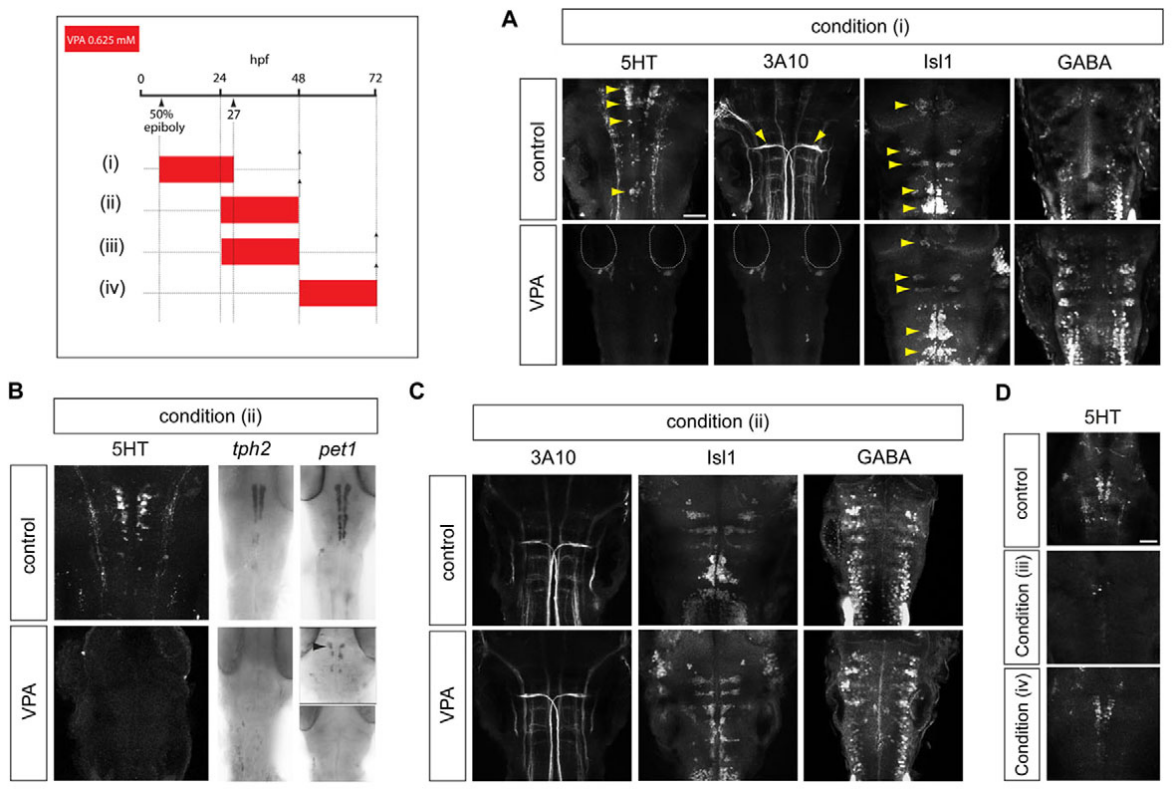
**Effect of valproate exposure on the differentiation of serotonergic and other brainstem neuronal subtypes.** The timing of drug treatment is indicated in this and all subsequent schematics by a coloured bar. Arrowheads mark the developmental stage of interest (hpf). Dashed lines with arrows indicate the developmental time point at which embryos were harvested. (A) Immunostaining for brainstem neuronal subtypes in 48 hpf zebrafish embryos exposed to VPA from 50% epiboly to 27 hpf [condition (i)] shows the failure of 5HT neuronal differentiation (*n*=22/22, *P*<0.0001), marked by 5HT (yellow arrowheads in control panel), and lack of Mauthner neurons (*n*=22/22), marked by the 3A10 anti-neurofilament monoclonal antibody (yellow arrowheads in control panel). Isl1^+^ motor neurons (yellow arrowheads in control and VPA panels) (*n*=10/10) and GABA-ergic neurons (*n*=10/10) persist. Scale bar: 50 μm. (B) Failure of serotonergic differentiation in embryos treated with VPA from 24-48 hpf [condition (ii)], as indicated by lack of 5HT (*n*=32/32, *P*<0.0001) and *Tph2* expression. *Pet1* (arrowhead) is severely reduced (*n*=3/15) or absent (*n*=12/15) in VPA-treated embryos. (C) Persistence of Mauthner, motor and GABA-ergic neurons in VPA treatment condition (ii). (D) At 72 hpf, embryos treated with VPA from 24-48 hpf [condition (iii)] show a limited recovery of 5HT expression (*n*=30/30). Treatment with VPA from 48-72 hpf [condition (iv)] does not affect serotonergic differentiation (*n*=30/30). hpf, hours post-fertilisation. Scale bar: 50 μm.

To assess the effect of VPA on brainstem development, we exposed zebrafish gastrulae at 50% epiboly to 0.625 mM VPA until 27 hours post-fertilisation (hpf), at which time the drug was removed and the embryos were allowed to develop until 48 hpf. Treatment from gastrulation was based on the heightened risk of teratogenicity in human infants exposed to VPA during the first trimester of pregnancy (Ornoy, 2009). Immunostaining for a range of hindbrain neuronal subtypes, specifically motor neurons, 5HT neurons, GABA-ergic neurons and Mauthner neurons, revealed a failure of 5HT neuronal differentiation marked by absence of 5HT expression (Fig. 1A). Additionally, Mauthner neurons, which express a neurofilament-associated antigen that is detected by the 3A10 monoclonal antibody, were also absent (Hatta, 1992; Schier et al., 1996) (Fig. 1A). Isl1-positive motor neurons and GABA-ergic neurons were present. The spatial distribution of motor neurons in VPA-treated embryos appeared subtly altered, suggesting migratory defects, but we did not pursue these changes further (Fig. 1A). Instead we focused on the striking serotonergic phenotype.

We speculated that exposure to VPA from gastrulation could affect common steps in the differentiation of multiple neuronal lineages. Therefore, we treated embryos with 0.625 mM VPA from 24-48 hpf and used appropriate markers to detect brainstem neuronal subtypes (Fig. 1B,C). Somatic motor, GABA-ergic and Mauthner neurons were still present (Fig. 1C), but there was a specific deficit of 5HT neuronal differentiation, marked by the absence of 5HT and *tph2* expression (Fig. 1B). Moreover, there was an absence or severe reduction of *pet1* expression, which indicates that VPA acts at a step proximal to or at the early stages of 5HT neuronal differentiation (Fig. 1B). Lower doses than this did not consistently lead to the loss of 5HT expression (see supplementary material Fig. S1).

Having established that brainstem 5HT expression was specifically lacking in embryos treated with VPA from 24 hpf, we addressed whether there was a delay in the differentiation of 5HT neurons. VPA was removed at 48 hours and embryos were analysed after a further 24 hours of incubation (Fig. 1D). There was limited recovery of 5HT expression at 72 hpf [mean number of 5HT neurons in controls=26.7±1.7 (s.d.), *n*=3; mean number of 5HT neurons in VPA-treated condition=1.8±1.3, *n*=4], which suggests that VPA does not merely retard the differentiation of 5HT neurons. We conclude that VPA specifically blocks the differentiation of 5HT neurons in embryos exposed to the drug between 24 and 48 hpf. This period coincides with the onset of differentiation of 5HT neurons between 25 and 30 hpf (Lillesaar et al., 2007). Because the post-mitotic differentiation of 5HT neurons is well underway from 48 hpf onwards (Lillesaar et al., 2007; McLean and Fetcho, 2004), we asked whether VPA could abolish 5HT expression after 48 hpf. VPA exposure between 48 hpf and 72 hpf had no effect on 5HT expression (Fig. 1D) (mean number of 5HT neurons=25.8±2.5, *n*=4). These data suggest that VPA acts on serotonergic progenitors, rather than on post-mitotic neurons.

To test whether specific neuronal subtypes are vulnerable to VPA application from 24 hpf because they only differentiate at or after 24 hpf, we extended the range of neuronal subtypes assayed. Cerebellar Purkinje neurons begin to differentiate from 3 dpf and are marked by expression of Parv7 (Bae et al., 2009). Wild-type embryos were incubated in VPA for a prolonged period, from 24 hpf to 4.5 dpf (see supplementary material Fig. S2). Immunostaining against Parv7 showed the persistence of this cell type in VPA-treated embryos (supplementary material Fig. S2). The differential sensitivity of 5HT and Purkinje neurons suggests that vulnerability to the effects of VPA is not linked directly to the timing of neuronal differentiation.

### Inhibition of Hdac1 by VPA accounts for the failure of 5HT neuronal differentiation

**Fig. 2.**
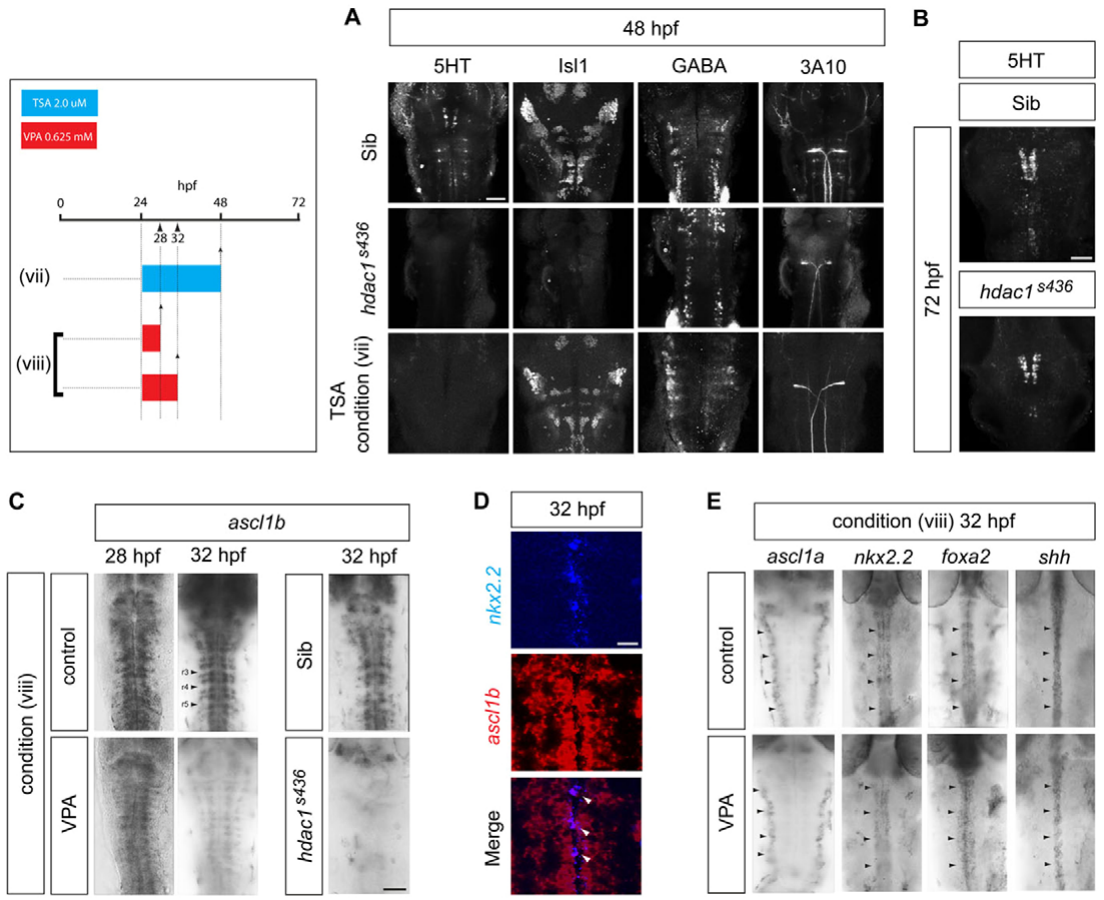
**Failure of serotonergic differentiation in VPA-treated embryos is mimicked by blockade of Hdac1 activity and is associated with downregulation of *ascl1b*.** (A) *hdac1* mutation or pharmacological blockade mimics the effect of VPA on the differentiation of brainstem neuronal subtypes. At 48 hpf, *hdac1^s436^* mutants lack 5HT neurons (*n*=15/15, *P*<0.0001) and show a severe depletion of Isl1^+^ motor neurons. Treatment with TSA from 24 to 48 hpf [condition (vii)] selectively affects 5HT expression (*n*=30/30, *P*<0.0001), but other neuronal subtypes are retained. Scale bar: 50 μm. (B) Partial recovery of 5HT neuronal differentiation in *hdac1^s436^* mutants at 72 hpf (*n*=14/14). Scale bar: 50 μm. (C) Short duration (8 hour) exposure to VPA from 24 hpf [condition (vi)] leads to the downregulation of *ascl1b* expression (*n*=15/15), and similar loss of *ascl1b* expression is observed in *hdac1^s436^* mutants (*n*=10/10). Rhombomeres (r) marked by arrowheads. Scale bar: 50 μm. (D) Double *in situ* hybridisation showing *ascl1b* expression in serotonergic progenitors, marked by expression of *nkx2.2*. Co-labelled cells are indicated by white arrowheads. Scale bar: 20 μm. (E) Short-duration VPA treatment [condition (viii)] does not affect the expression of the paralogous gene *ascl1a*, or other progenitor-expressed determinants of serotonergic fate, *nkx2.2*, *foxa2* or *shh* (arrowheads).

Next, we sought to identify the molecular pathway targeted by VPA in blocking 5HT neuronal differentiation. Previous studies have shown both *in vivo* and *in vitro* that VPA is an inhibitor of HDACs at therapeutic concentrations (Göttlicher et al., 2001; Gurvich et al., 2005; Kook et al., 2003; Phiel et al., 2001; Tremolizzo et al., 2002; Yildirim et al., 2003). To test the involvement of Hdac1, we used a zebrafish *hdac1* mutant line, *hdac1^s436^*, that was previously isolated in a forward genetic screen (Noël et al., 2008). Immunostaining of *hdac1^s436^* mutants revealed a lack of 5HT expression in the brainstem at 48 hpf (Fig. 2A). However, by 72 hpf there was a partial recovery of 5HT neuronal differentiation (Fig. 2B) that was more complete than in VPA-treated embryos at the same stage (Fig. 1D, middle panel) [mean number of 5HT neurons in control siblings (sibs)=31±2.5, *n*=4; mean number of 5HT neurons in mutants=25.3±5.4, *n*=4]. Apart from a severe reduction in the differentiation of somatic motor neurons, marked by Isl1 expression, which has been reported previously (Cunliffe, 2004), other neuronal subtypes, GABA-ergic and Mauthner neurons, appeared intact (Fig. 2A).

To further test the involvement of HDACs in 5HT neuron production, wild-type embryos were treated with the HDAC inhibitor trichostatin A (TSA), a potent inhibitor of class I and class II HDACs (Yoshida et al., 1990). Pharmacological blockade of HDACs with TSA from 24-48 hpf recapitulated the effect of VPA treatment, and resulted in loss of 5HT expression at 48 hpf, with preservation of somatic motor, GABA-ergic and Mauthner cell differentiation (Fig. 2A). This suggests that the *hdac1^s436^* neuronal phenotype differs from the effect of VPA most likely because of differences in the timing of Hdac1 inactivation. Moreover, the closely similar phenotypes that result from *hdac1* mutation and VPA treatment are consistent with the idea that VPA blocks 5HT neuronal differentiation by inhibiting Hdac1.

### VPA downregulates expression of the proneural gene *ascl1b* in serotonergic progenitors through a mechanism consistent with Hdac1 inhibition

Next we examined the expression of transcription factors that specify serotonergic progenitors. We reasoned that, because 5HT neurons are absent, any disruption of serotonergic fate determinants might occur before the first 5HT neurons are born. We therefore exposed 24 hpf embryos to VPA and analysed gene expression by *in situ* hybridisation 8 hours later. There was a striking reduction of *ascl1b* expression by 32 hpf that was not apparent at 28 hpf, just 4 hours earlier (Fig. 2C). Colocalisation of *ascl1b* and *nkx2.2* in the hindbrain by fluorescent double *in situ* hybridisation confirmed the expression of *ascl1b* in serotonergic progenitors (Fig. 2D). Expression of the related gene *ascl1a* and other fate determinants, *nkx2.2* and *foxa2*, and the signalling molecule *shh* were unchanged, which implies that the loss of *ascl1b* expression is not due to depletion of progenitors (Fig. 2E).

Further evidence implicating Hdac1 as the mediator of the effects of VPA on 5HT neurons is that, in *hdac1^s436^* mutants, expression of *ascl1b* is also severely reduced, a finding that has been reported previously in a different *hdac1*-null mutant line (Fig. 2C) (Cunliffe, 2004).

In contrast to the downregulation of *ascl1b*, another bHLH proneural gene, *ptf1a*, which is required for the generation of cerebellar Purkinje neurons (Hori et al., 2008), was not decreased by VPA. *Ptf1a* expression in the cerebellar primordium is first detected at 48 hpf (Kani et al., 2010). We monitored *ptf1a* expression by *in situ* hybridisation for *ptf1a* transcripts in wild-type zebrafish embryos and by an eGFP reporter in a transgenic *ptf1a-egfp* line at 52 hpf in embryos continuously exposed to VPA from 24 hpf (see supplementary material Fig. S2) (Pisharath et al., 2007). The persistent *ptf1a* expression suggests that proneural genes are differentially sensitive to VPA and this is consistent with the continued differentiation of Purkinje neurons after VPA exposure (see supplementary material Fig. S2).

### Notch signalling represses *ascl1b* in the presence of VPA

VPA has previously been reported to upregulate Notch signalling in a variety of systems (Greenblatt et al., 2007; Stockhausen et al., 2005). In zebrafish embryos this effect seems likely to be mediated by Hdac1 (Cunliffe, 2004). One possibility, therefore, was that Hdac1 was inhibiting the Notch pathway and thus decreasing the Notch-mediated inhibition of *ascl1b* via Her genes, which are the major effectors of Notch signalling in zebrafish (Cunliffe, 2004; Louvi and Artavanis-Tsakonas, 2006). A second possible mechanism is through direct upregulation of *ascl1b* by Hdac1 (Cunliffe, 2004; Harrison et al., 2011; Wang et al., 2009). Finally, Hdac1 repression of specific members of the Her family, in particular *her6*, which in turn represses *ascl1b*, is a third possibility (Cunliffe, 2004; Harrison et al., 2011).

**Fig. 3.**
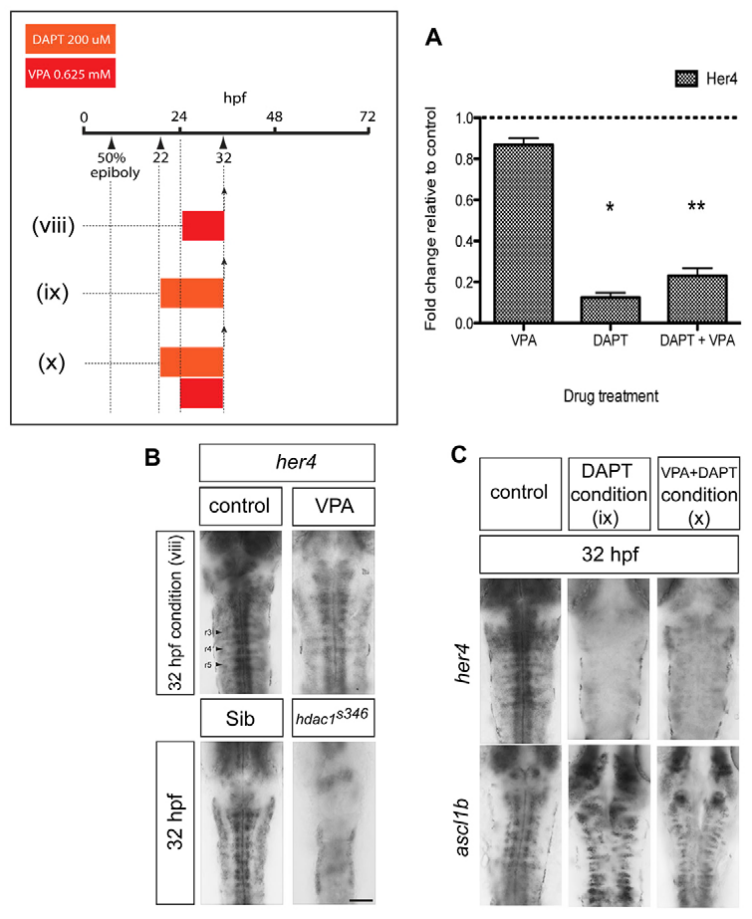
**VPA treatment exposes cryptic transcriptional repression of *ascl1b* by basal levels of Notch signalling.** (A) Quantitative reverse-transcriptase PCR showing that VPA mildly reduces *her4* expression, whereas DAPT strongly reduces *her4* expression to between 12% and 23% of control levels (*P=0.017, **P=0.001). (B) Short duration (8 hour) treatment with VPA [condition (viii)] does not upregulate *her4* expression at 32 hpf (*n*=12/12). Downregulation of *her4* expression in *hdac1^s436^* mutant hindbrain at 32 hpf (*n*=5/5). Scale bar: 50 μm. (C) Concomitant blockade of Notch signalling using the γ-secretase inhibitor DAPT prevents the downregulation of *ascl1b* by VPA. Loss of *her4* expression in DAPT-treated embryos [conditions (ix) and (x)] (upper panels) (*n*=35/35). Persistence of *ascl1b* expression in embryos treated with VPA and DAPT [condition (x)] (*n*=23/23).

*her4* is a direct target of Notch signalling that is widely used as a readout of Notch activity (Takke and Campos-Ortega, 1999; Takke et al., 1999; Yeo et al., 2007). The downregulation of *ascl1b* in embryos following an 8-hour treatment with VPA from 24 hpf (Fig. 2C) was not accompanied by obvious elevation of *her4* expression (Fig. 3A,B). Quantitative reverse-transcriptase PCR confirmed that there was a modest decrease in *her4* expression relative to untreated control embryos (Fig. 3A). *her6* is also a target of the Notch pathway and to date is the only Her gene reported to undergo negative regulation by Hdac1 (Cunliffe, 2004). *In situ* hybridisation for *her6* and a panel of additional Her family members in wild-type embryos similarly treated with VPA also showed no difference in expression compared with controls (data not shown).

To investigate the involvement of the Notch pathway in *ascl1b* repression by VPA, we inhibited Notch signalling over the same period and determined whether VPA could still repress *ascl1b*. Embryos at 22 hpf were treated with a small-molecule inhibitor of Notch signalling, the γ-secretase inhibitor DAPT, which is known to have potent activity *in vivo* in zebrafish (Geling et al., 2002; Imbimbo, 2008). DAPT treatment alone from 22-32 hpf effectively downregulated *her4* expression, whereas *ascl1b* expression was maintained (Fig. 3A,C). Strikingly, following the addition of VPA at 24 hpf, *her4* remained repressed and *ascl1b* expression persisted (Fig. 3A,C). Therefore, the repression of *ascl1b* by short-term exposure to VPA is prevented by concomitant blockade of Notch signalling, which implies that a basal level of Notch signalling represses *ascl1b* when Hdac1 activity is blocked.

To confirm that the repression of *Ascl1b* by VPA is not due to increased Notch signalling secondary to blockade of Hdac1 function, we analysed *her4* expression in *hdac1^s436^* mutants at 32 hpf (Fig. 3B). There was a marked reduction of *her4* expression in mutant hindbrains, indicative of reduced Notch signalling (Fig. 3B). Therefore, VPA inhibition of Hdac1 does not repress *ascl1b* through the upregulation of Notch signalling, or via Hdac1-mediated repression of *her4* or *her6*. Instead, Hdac1 acts independently of Notch activity to regulate *ascl1b*. Moreover, a parallel, tonic level of Notch signalling was sufficient to repress *ascl1b* transcription when Hdac1 was inhibited by VPA.

### Hdac1 and Notch have opposing effects on *ascl1b*

**Fig. 4.**
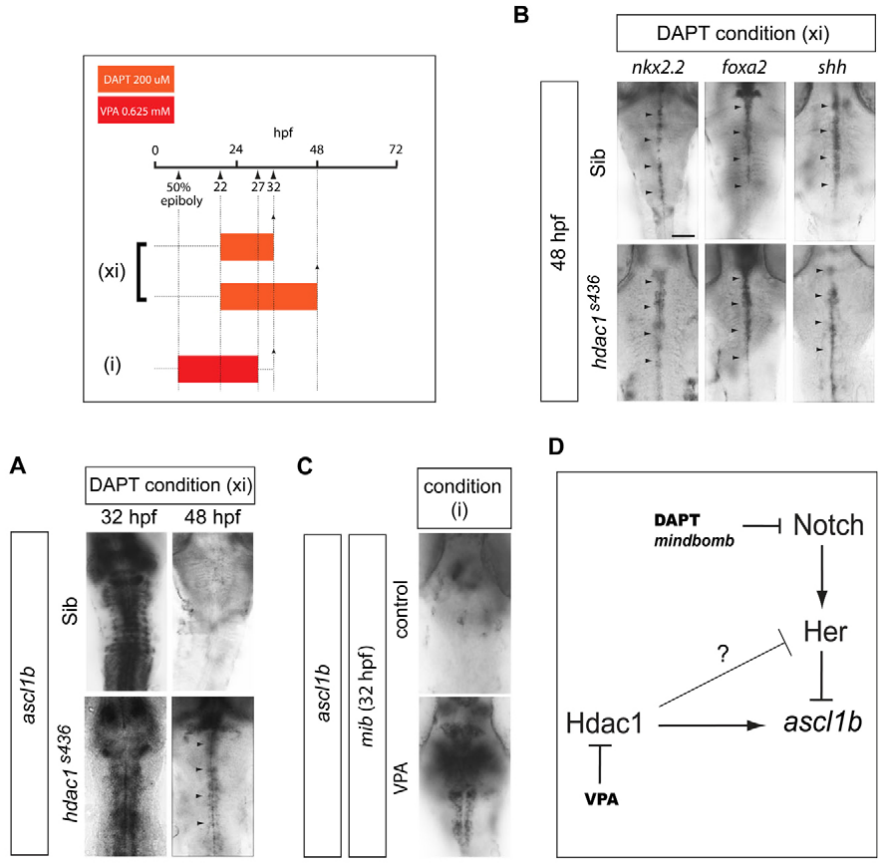
***Ascl1b* transcriptional regulation by opposing Hdac1 and Notch activities.** (A) Blockade of Notch signalling by DAPT in *hdac1^s436^* mutants [condition (xi)] results in the recovery of *ascl1b* expression at 32 hpf and 48 hpf (arrowheads) (*n*=15/15 for both developmental stages combined). Arrowheads indicate the expression domain of *ascl1b*. (B) DAPT treatment of *hdac1^s436^* mutants [condition (xi)] does not affect the expression of *nkx2.2* (*n*=7/7), *foxa2* (*n*=8/8) and *shh* (*n*=6/6) (arrowheads) at 48 hpf. Arrowheads indicate the expression domain of the respective genes in the ventral midline of the embryo. Scale bar: 50 μm. (C) VPA treatment of embryos on the Notch signalling mutant *mindbomb* (*mib*) background [condition (i)] is associated with recovery of *ascl1b* expression (*n*=11/11). (D) Model of transcriptional regulation of *ascl1b* by Hdac1 and the Notch pathway. Solid arrows indicate direct positive regulation. Lines with an orthogonal bar represent inhibition. The inhibitory arrow with a question mark above it, from Hdac1 to Her, takes account of the possibility that another Her gene (or another transcription factor of unknown identity) that represses *ascl1b* is in turn repressed by Hdac1.

To confirm the parallel roles for Hdac1 and Notch signalling, we tested whether DAPT could restore *ascl1b* expression in *hdac1^s436^* mutants. *hdac1^s436^* mutants and sibs were treated with DAPT from 22 hpf (Fig. 4A). At 32 hpf, sibs treated with DAPT showed strong expression of *ascl1b*, which was virtually absent by 48 hpf presumably because of depletion of the progenitor pool caused by the blockade of Notch signalling. Remarkably, DAPT restored *ascl1b* expression in *hdac1^s436^* mutants, and expression was maintained at least up to 48 hpf (compare Fig. 2C and Fig. 4A). The other progenitor markers, *nkx2.2*, *foxa2* and *shh*, were not altered by DAPT in either sibs or *hdac1^s436^* embryos at 48 hpf (Fig. 4B). These data confirm that Hdac1 and Notch exert positive and negative regulatory effects, respectively, on *ascl1b*. Given the finding that DAPT could prevent the repression of *ascl1b* by VPA, the expression of *ascl1b* was then analysed in a Notch-signalling mutant, *mindbomb* (*mib*). These mutants lack an E3 ubiquitin ligase, which is crucial for Notch signalling (Itoh et al., 2003; Schier et al., 1996). Consistent with the data from chemical inhibition of Notch signalling, the expression of *ascl1b* was restored in 32 hpf *mib* mutants treated with VPA from 50% epiboly to 27 hpf (Fig. 4C).

### Failure of 5HT neuronal differentiation in VPA-treated embryos is due to repression of *ascl1b*

**Fig. 5.**
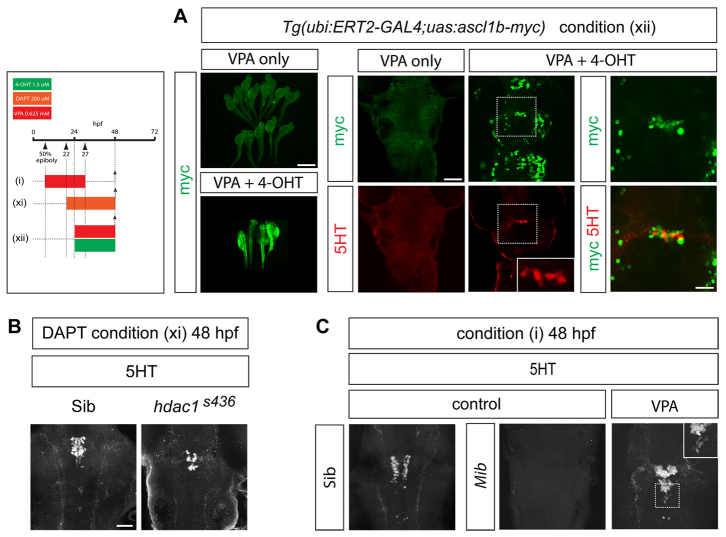
**Ascl1b is sufficient to rescue serotonergic differentiation in VPA-treated embryos.** (A) Mis-expression of Myc-tagged Ascl1b in VPA-treated transgenic embryos rescues 5HT neuronal differentiation. A stable transgenic line expressing *ERT2-GAL4* under the control of the *ubiquitin* (*ubi*) promoter was injected at the one-cell stage with plasmid DNA encoding Ascl1b-Myc under the control of *UAS*. Embryos were treated with VPA with or without 4-hydroxytamoxifen (4-OHT) [condition (xii)]. Myc immunostaining in shown in green. The upper and lower left panels show low-power views (scale bar: 500 μm) of Myc-immunostained zebrafish embryos treated with VPA in the absence (upper panel) or presence (lower panel) of 4-OHT. Addition of 4-OHT leads to widespread expression of Ascl1b-Myc (bottom left panel). Panels to the right show high-power views (scale bar: 50 μm) of Myc (green) and 5HT (red) immunostaining in the hindbrain of transgenic embryos. Embryos treated with VPA alone fail to express 5HT (*n*=51/51). Addition of 4-OHT and induction of Ascl1b-Myc expression rescues 5HT expression in 8/51 VPA-treated embryos (*P*=0.0058). Upper right and bottom right panels are high-power (scale bar: 20 μm) views of the boxed areas in the panels immediately to the left. The bottom, middle panel inset shows a higher-power view of the 5HT-expressing cells in the boxed area. The bottom right panel shows merged channels representing Ascl1b-Myc (green) and 5HT (red) immunostaining. (B) In *hdac1^s436^* mutants treated with DAPT [condition (xi)], there is a rescue of 5HT neuronal differentiation at 48 hpf (*n*=22/22, *P*<0.0001). Scale bar: 50 μm. (C) On the *mib* mutant background there is a recovery of 5HT neurons at 48 hpf in VPA-treated embryos [condition (i)] (*n*=12/12, *P*=0.0002). Inset shows a high-power image of the boxed region.

Finally, we asked whether replacement of *ascl1b* was sufficient to rescue 5HT neuronal differentiation in VPA-treated embryos. To test this, a plasmid encoding a Myc-tagged full-length Ascl1b (*ascl1b-myc*) under the control of *UAS* was injected into a stable transgenic zebrafish line, *ubi:ERT2-GAL4* (Gerety et al., 2013), which allows the regulation of *ascl1b-myc* by 4-hydroxytamoxifen (4-OHT). In control embryos treated with VPA alone, Ascl1b-Myc could not be detected by immunostaining for Myc antigen (Fig. 5A) and 5HT-expressing neurons were absent. Addition of 4-OHT resulted in strong and widespread expression of Ascl1b-Myc and the rescue of 5HT neuronal differentiation in 16% of embryos (Fig. 5A, *P*=0.0058).

If repression of *ascl1b* is the reason for the failure of 5HT neuronal differentiation upon VPA exposure, then restoration of *ascl1b* expression in *hdac1^s436^* and *mib* mutants should be sufficient to rescue 5HT expression. Indeed, immunostaining of DAPT-treated *hdac1^s436^* embryos at 48 hpf revealed the rescue of 5HT expression in the raphe (Fig. 5B). We also exposed *mib* mutants to VPA from gastrulation (50% epiboly) to 27 hpf, and then incubated the embryos in normal medium until 48 hpf, at which time they were fixed and immunostained for 5HT. We had already established that an identical VPA regime abolishes 5HT expression on a wild-type background (Fig. 1A). Untreated *mib* mutants lacked 5HT neurons but, remarkably, 5HT neurons were generated in *mib* mutant embryos exposed to VPA (Fig. 5C). These findings confirmed that the loss of 5HT expression in VPA-treated embryos was not due to neuronal death, but instead was due to a block in their differentiation.

## DISCUSSION

Using a zebrafish model of FVS, we have identified a molecular mechanism to explain the loss of 5HT neurons, which have been implicated in the neuropsychiatric manifestations of FVS. Two different VPA treatment regimes caused the failure of serotonergic differentiation, and the late-dosing regime (24-48 hpf) seemed to have greater selectivity for the serotonergic system than the early-dosing schedule (50% epiboly to 27 hpf). There is wide variation in the teratogenic dose of VPA in human and animal studies (Christensen et al., 2013; Ornoy, 2009), which makes direct comparisons of dosing regimes between different model organisms and with humans exposed to VPA *in utero* difficult to interpret. Nevertheless, the altered serotonergic differentiation in this zebrafish model is reminiscent of similar deficits that are found in rodent models, which correlate with autism-like behavioural abnormalities (Dufour-Rainfray et al., 2010; Dufour-Rainfray et al., 2011).

Recovery of *ascl1b* expression in VPA-treated *hdac1^s436^* or *mib* mutant embryos is associated with rescue of 5HT neuronal differentiation. However, direct replacement of Ascl1b is sufficient to rescue 5HT expression in only 16% of embryos. Suboptimal timing or level of the ectopically expressed Ascl1b expression might explain the lower efficiency. Alternatively, non-physiological, persistent high-level expression of Ascl1b in post-mitotic neurons might account for the low rate of rescue (Cai et al., 2000). Nevertheless, taken together these findings indicate that the failure of 5HT neuronal differentiation in our model of FVS is most probably due to the loss of *ascl1b* expression. We cannot, however, exclude the possibility that VPA disrupts the expression of other, unknown, genes that are crucial for 5HT neuronal differentiation. We anticipate that the finding of downregulated expression of *ascl1b* in our zebrafish model will prompt closer analysis of rodent FVS models for changes in the expression of the mammalian ortholog *Ascl1* in serotonergic progenitors. *Ascl1* is known to be required for neuronal-subtype specification, which is separable from its better-known proneural function (Goridis and Brunet, 1999). In mammals, a requirement for *Ascl1* in serotonergic differentiation has previously been demonstrated, including for the expression of 5HT itself, and this subtype-specification function cannot be substituted by other proneural genes (Jacob et al., 2009; Parras et al., 2002; Pattyn et al., 2004). Additionally, *Ascl1* has been shown to be essential for the acquisition of noradrenergic traits in all mammalian central and peripheral neurons (Blaugrund et al., 1996; Guillemot et al., 1993; Hirsch et al., 1998). At least in part, *Ascl1* regulates neurotransmitter phenotype in noradrenergic neurons in collaboration with *Phox2* genes (Pattyn et al., 2000). However, *Phox2* genes are not expressed in the mammalian serotonergic lineage. Instead, *Ascl1* regulation of the zinc-finger transcription-factor-encoding gene *Insm1* in post-mitotic serotonergic precursors is a crucial component of the mammalian serotonergic transcriptional programme (Jacob et al., 2009).

Using the HDAC inhibitor TSA and an *hdac1* mutant line, we have provided evidence that the effect of VPA on serotonergic differentiation is mediated through Hdac1 inhibition. These findings are consistent with previous reports that VPA inhibits HDACs *in vivo* (Gurvich et al., 2005). Moreover, Hdac1 is one of the key molecular factors that regulate the transcription of *ascl1b* and these data are consistent with the previously identified positive regulatory role of Hdac1 (Cunliffe, 2004; Harrison et al., 2011). The regulation of *ascl1b* might be direct given that Hdac1 binds to the *ascl1b* promoter (Harrison et al., 2011). It has been proposed that Hdac1 promotes activation of *ascl1b* directly by participating in cycles of deacetylation and histone-acetyltransferase-dependent acetylation of transcription-unit-associated histones, and/or by maintaining the *ascl1b* promoter in a transcriptionally poised state (Wang et al., 2009). Nevertheless, we do not rule out the possibility that Hdac1 functions indirectly by inhibiting the expression of a transcriptional repressor of *ascl1b* (Fig. 4D).

A second transcriptional regulator of *ascl1b* that is unmasked by VPA is repressive regulation by the Notch pathway. It was shown previously that VPA upregulates Notch signalling and this is associated with increased expression of Notch effector genes (Greenblatt et al., 2007; Stockhausen et al., 2005). Therefore, we expected that repression of *ascl1b* by VPA might be mediated by enhanced Notch signalling. However, short-duration VPA treatment is sufficient to downregulate *ascl1b* without concomitantly enhancing Notch signalling. Consistent with this, the expression of a panel of Her genes, including *her4*, a target of Notch signalling, and *her6*, are not appreciably altered under these conditions. Nevertheless, our findings using chemical and genetic blockade of Notch function demonstrate that parallel Notch signalling participates in the VPA-mediated downregulation of *ascl1b* (Fig. 4D). Importantly, only a basal level of Notch signalling is required for VPA downregulation of *ascl1b*. Together, our findings lead to a revised view of *ascl1b* regulation based on a derepression model, because removal of the basal inhibitory Notch input when Hdac1 function is blocked by VPA is sufficient for *ascl1b* transcription.

The regulatory relationship between Hdac1 and the Notch pathway in the control of *ascl1b* expression reported in a previous study (Cunliffe, 2004) provided an opportunity to evaluate further whether VPA blockade of serotonergic differentiation occurs via Hdac1 inhibition. In *hdac1* mutants, we found that *her4* expression is reduced (Fig. 3B). Our result differs from the latter study that proposed increased Notch signalling in *hdac1* mutants based on enhanced *her6* expression (Cunliffe, 2004). A possible explanation for the difference is that *her6* expression in *hdac1* mutants is developmental-stage-dependent. The loss of *ascl1b* expression in *hdac1^s436^* mutants and in VPA-treated embryos (Fig. 2C) occurs in the absence of enhanced Notch pathway activity during the period of treatment. This is consistent with Hdac1 inhibition as the salient *in vivo* mechanism of VPA. Moreover, these data are also consistent with direct regulation of *ascl1b* by Hdac1.

Because VPA has been reported to have a variety of modes of action, identifying which of these mechanisms is relevant *in vivo* for the behavioural manifestations of FVS is challenging. This is an important question, however, because identification of the molecular mechanism has potential therapeutic significance. The involvement of Hdac1 in the serotonergic deficit in FVS strongly suggests that an epigenetic mode of action could mediate at least some of the behavioural manifestations of FVS. This is not an isolated finding, because epigenetic contributions to other ASDs have been noted. Methyl-CpG-binding protein 2 (*MECP2*), which is implicated as the cause of the ASD-associated disease Rett syndrome contains a transcriptional repression domain, which physically interacts with the transcriptional co-repressor Sin3A (Amir et al., 1999). In turn, Sin3A recruits HDAC1 and HDAC2, which are believed to mediate transcriptional repression by MECP2 (Jones et al., 1998; Nan et al., 1998). More broadly, epigenetic modifications seem to regulate autism susceptibility; for example, autism is associated with duplications of 15q11-13, which is an imprinted region of the genome where DNA methylation status is associated with Prader-Willi syndrome and Angelman syndrome (Dykens et al., 2011). In conclusion, epigenetic regulation of gene expression seems to be an emerging point of convergence for environmental agents and hereditary factors in ASD pathogenesis.

## MATERIALS AND METHODS

### Zebrafish strains and husbandry

Zebrafish embryos were staged according to hpf and morphological criteria (Kimmel et al., 1995). *mib* mutant embryos were obtained by incrosses of heterozygote *mib* zebrafish. They were identified by the irregular appearance of the hindbrain, markedly reduced hindbrain ventricle and loss of early somite boundaries (Stickney et al., 2000; van Eeden et al., 1996). *hdac1^s436^* mutants were generated by incrosses of heterozygote *hdac1^s436^* zebrafish (Noël et al., 2008). Mutants were identified by their smaller CNS, narrow anterior rhombomeres, reduced midbrain ventricle, cleft eyes, retinal hypopigmentation, curled shape and, at 48 hpf, additionally by absent pectoral fins (Cunliffe, 2004). *ubi:ERT2-GAL4* transgenic zebrafish were generated as described (Gerety et al., 2013).

### Transgenic constructs and transient transgenic zebrafish

DNA encoding Ascl1b tagged with six copies of Myc at the C-terminus was made by gene synthesis (Genwiz). *ascl1b-myc* was subcloned into a 5× *UAS* plasmid containing a miniTOL2 backbone to facilitate genomic integration in zebrafish (Balciunas et al., 2006). Transient transgenic embryos were generated by co-injecting 10-20 pg of *UAS:ascl1b-myc* plasmid DNA with 25 pg of *tol2* transposase mRNA into one-cell-stage embryos obtained by intercrossing *UBI:ERT2-GAL4* transgenic zebrafish.

### Reverse transcriptase PCR (RT-PCR) and primer sequences

Between 30 and 40 embryos at 32 hpf were used for each condition and experiments were performed in triplicate. At the end of the period of drug treatment embryos were placed in Trizol, homogenised, chloroform was added, the sample was centrifuged at 4°C, glycogen and isopropanol were added, the sample was then centrifuged again at 4°C, 75% ethanol was added, centrifugation at 4°C was repeated and the pellet was re-suspended in water. RNA purification was performed using the RNeasy Micro Kit (Qiagen) according to the manufacturer’s instructions. cDNA synthesis was performed using the Superscript First-Strand Synthesis System for RT-PCR (Invitrogen), according to the manufacturer’s instructions. Quantitative RT-PCR was performed using the 7900HT Fast Real-Time PCR System (Applied Biosystems).

The following primers were used for RT-PCR: *her4* 5′-AGCAGCAGCCCGACTCCAGA-3′, 5′-GCTGACGGCCTCCTGCACAC-3′; *beta-actin2* 5′-CGAGCTGTCTTCCCATCCA-3′, 5′-TCACCAACGT -AGCTGTCTTTCTG-3′. Data was analysed using the Student’s *t*-test.

### Immunohistochemistry and *in situ* hybridisation

For immunofluorescence and *in situ* hybridisation, embryos were fixed in 4% paraformaldehyde (PFA) either for 2-3 hours at room temperature or overnight at 4°C. For immunofluorescence, embryos were rinsed three times in 0.1% Tween in phosphate buffered saline (PBST), incubated for 20 minutes in 0.01 mg/ml proteinase K (Roche) in PBST/0.2% BSA/2% heat-inactivated goat serum solution (PBT), post-fixed in PFA, washed three times in PBT, blocked in PBT containing 1% DMSO, 0.5% Triton-X and 1% BSA (PBDT), and then incubated for 24 hours at 4°C in primary antibody in PBST. The following day, embryos were washed four times in PBT, blocked again in PBDT and then incubated in secondary antibody overnight in PBDT. Further washes in PBT were performed the next day, and embryos were then transferred to 70% glycerol and mounted under coverslips for viewing. Immunofluorescence staining was visualised by confocal microscopy (Leica TCS SP2). For single and double *in situ* hybridisation, embryos were processed as described (Lauter et al., 2011). Fluorescent signals were developed with Fast Blue and Fast Red. Differences between embryos were analysed using Fisher’s exact test.

The following antibodies were used: rabbit anti-5HT (1/500, Millipore), rabbit anti-GABA (1/500, Sigma), 3A10 mouse anti-neurofilament (1/25, DSHB), mouse anti-Parvalbumin7 (1/1000, gift from Dr Masahiko Hibi, Nagoya University, Japan), 4D5 mouse anti-Isl1 (1/45, DSHB), mouse anti-myc (1/500, Santa Cruz). The following riboprobes were used: *foxa2* (Strähle et al., 1993), *nkx2.2* (Barth and Wilson, 1995), *shh* (Krauss et al., 1993), *her4* (Takke et al., 1999), *her6*, *tph2* (also called *tphR*) (Teraoka et al., 2004), *ascl1a*, *ascl1b* (Allende and Weinberg, 1994), *ptf1a* (Zecchin et al., 2004). *pet1* (IMAGE ID: 7000463) was subcloned from p-Express1 into pBluescript, then linearised with *Eco*RI (Roche) and transcribed using T7 Polymerase (Promega).

### Drugs

Embryos were dechorionated manually prior to drug treatment. Sodium valproate (Sigma) was dissolved in water as a 2 M stock solution and was used at a final concentration of 0.625 mM. This dose is comparable to doses found to inhibit HDACs *in vivo* in other studies (Gurvich et al., 2005). Lower doses did not consistently abolish 5HT expression in the hindbrain (see supplementary material Fig. S1). DAPT (Calbiochem) was dissolved in DMSO as a 46 mM stock solution and used at 200 μM final concentration, which is similar to doses previously reported to block Notch signalling in zebrafish (Geling et al., 2002). A 5 mM TSA solution in DMSO (Sigma) was diluted to a final concentration of 2 μM in fish water prior to use, which is comparable to doses used *in vivo* to inhibit HDACs (Gurvich et al., 2005). 4-OHT (Sigma) was dissolved in ethanol at 12.5 mg/ml. Dilutions of 4-OHT were made to 1.5 μM in standard fish water just prior to use. In all drug conditions, controls were treated with an equivalent amount of vehicle diluted in fish water. Stock solutions of all these drugs were stored at −20°C.

## Supplementary Material

Supplementary Material
