# Supplementary material for: Valproic acid silencing of ascl1b/Ascl1 results in the failure of serotonergic differentiation in a zebrafish model of fetal valproate syndrome
Source: Dis Model Mech. 2013 Oct 17;7(1):107–17. doi: 10.1242/dmm.013219 (PMC3882053; doi:10.1242/dmm.013219)
Supplement: Supplementary Material [file supp_7_1_107__index.html]

Valproic acid silencing of ascl1b/Ascl1 results in the failure of serotonergic differentiation in a zebrafish model of fetal valproate syndrome — Supplementary Material 

# Valproic acid silencing of *ascl1b/Ascl1* results in the failure of serotonergic differentiation in a zebrafish model of fetal valproate syndrome

## DMM013219 Supplementary Material

**Files in this Data Supplement:**

- **Supplementary Material PDF**
