# Supplementary material for: Valproic acid silencing of ascl1b/Ascl1 results in the failure of serotonergic differentiation in a zebrafish model of fetal valproate syndrome
Source: Dis Model Mech. 2013 Oct 17;7(1):107–17. doi: 10.1242/dmm.013219 (PMC3882053; doi:10.1242/dmm.013219)
Supplement: Supplementary Material [file supp_013219_DMM013219.pdf]

SUPPLEMENTARY MATERIAL

Supplementary Figures

Figure S1 Inconsistent effect of 0.3 mM valproate on 5HT expression in the zebrafish brainstem. Embryos were treated with VPA from 24 to 48 hpf at which time they were harvested and immunostained for 5HT. Control 5HT expression is shown on the right. VPA treatment inconsistently abolished the expression of 5HT (middle and left panels). Representative embryos are shown. Arrow shows residual 5HT neurons in a valproate treated embryo. Quantification of the number of embryos in which 5HT expression is either absent or present is shown in the panels as a proportion of the total number of embryos.

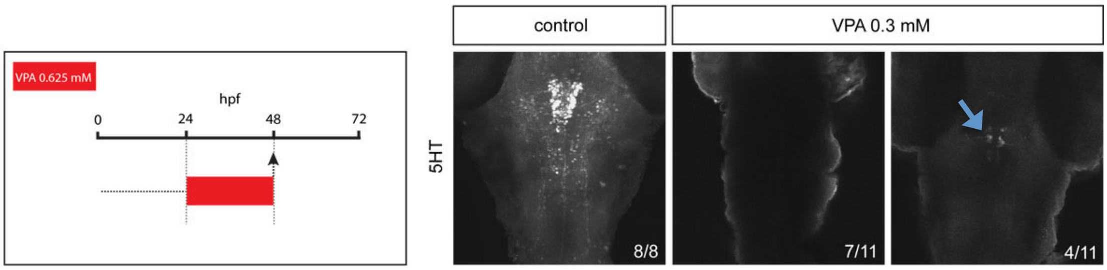

Figure S2 Cerebellar Purkinje cell differentiation appears intact in valproate treated embryos.

A. Lateral views of differentiating Purkinje neurons (blue arrowheads) immunostained with anti-Parv7 antibody at 4.5 dpf in control and VPA treated embryos [condition (v)].

B. Lateral views of 52 hpf embryos showing expression of *ptf1a* in the cerebellar anlage of control and VPA treated embryos (dotted circles). Dorsal views of GFP expression in 52 hpf *ptfa1-egfp* transgenic reporter embryos. The expression of *ptf1a* transcript and the GFP reporter is not downregulated by VPA treatment from 24 hpf [condition (vi)].

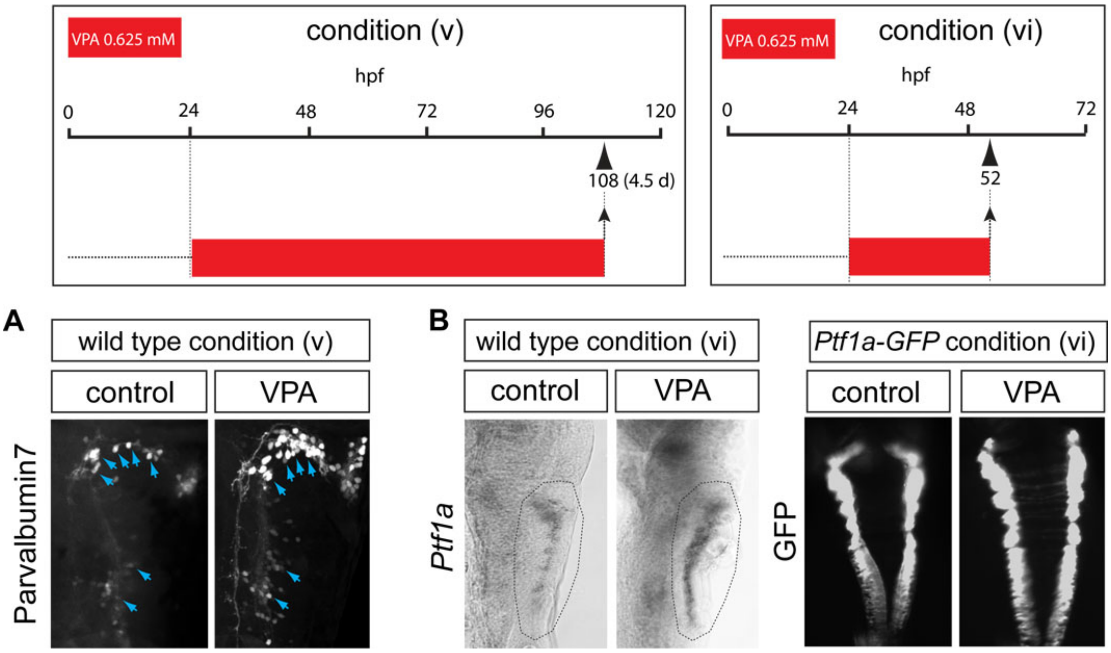

VPA 0.625 mM
condition (v)
hpf
0
24
48
72
96
120
108 (4.5 d)
VPA 0.625 mM
condition (vi)
hpf
0
24
48
72
52
A
wild type condition (v)
control
VPA
Parvalbumin7
B
wild type condition (vi)
control
VPA
Ptf1a
Ptf1a-GFP condition (vi)
control
VPA
GFP
